# Models and Techniques to Study Aortic Valve Calcification *in Vitro*, *ex Vivo* and *in Vivo*. An Overview

**DOI:** 10.3389/fphar.2022.835825

**Published:** 2022-06-02

**Authors:** Maria Bogdanova, Arsenii Zabirnyk, Anna Malashicheva, Daria Semenova, John-Peder Escobar Kvitting, Mari-Liis Kaljusto, Maria del Mar Perez, Anna Kostareva, Kåre-Olav Stensløkken, Gareth J Sullivan, Arkady Rutkovskiy, Jarle Vaage

**Affiliations:** ^1^ Department of Molecular Medicine, Institute of Basic Medical Sciences, University of Oslo, Oslo, Norway; ^2^ Department of Research and Development, Division of Emergencies and Critical Care, Oslo University Hospital, Oslo, Norway; ^3^ Institute of Cytology, Russian Academy of Sciences, Saint Petersburg, Russia; ^4^ Department of Cardiothoracic Surgery, Oslo University Hospital, Oslo, Norway; ^5^ Sanifit Therapeutics, Palma de Mallorca, Spain; ^6^ Almazov National Medical Research Centre, Saint Petersburg, Russia; ^7^ Department of Woman and Children Health, Karolinska Institute, Stockholm, Sweden; ^8^ Norwegian Center for Stem Cell Research, Oslo University Hospital and University of Oslo, Oslo, Norway; ^9^ Institute of Immunology, Oslo University Hospital, Oslo, Norway; ^10^ Hybrid Technology Hub - Centre of Excellence, Institute of Basic Medical Sciences, University of Oslo, Oslo, Norway; ^11^ Department of Pediatric Research, Oslo University Hospital, Oslo, Norway; ^12^ Department of Pulmonary Diseases, Oslo University Hospital, Oslo, Norway; ^13^ Institute of Clinical Medicine, University of Oslo, Oslo, Norway

**Keywords:** aortic valve, interstitial cells, endothelial cells, calcification, animal models, calcified aortic valve disease, imaging

## Abstract

Aortic valve stenosis secondary to aortic valve calcification is the most common valve disease in the Western world. Calcification is a result of pathological proliferation and osteogenic differentiation of resident valve interstitial cells. To develop non-surgical treatments, the molecular and cellular mechanisms of pathological calcification must be revealed. In the current overview, we present methods for evaluation of calcification in different *ex vivo*, *in vitro* and *in vivo* situations including imaging in patients. The latter include echocardiography, scanning with computed tomography and magnetic resonance imaging. Particular emphasis is on translational studies of calcific aortic valve stenosis with a special focus on cell culture using human primary cell cultures. Such models are widely used and suitable for screening of drugs against calcification. Animal models are presented, but there is no animal model that faithfully mimics human calcific aortic valve disease. A model of experimentally induced calcification in whole porcine aortic valve leaflets *ex vivo* is also included. Finally, miscellaneous methods and aspects of aortic valve calcification, such as, for instance, biomarkers are presented.

## 1 Introduction

Calcific aortic valve disease (CAVD) is a slowly progressing disorder starting with non-symptomatic thickening and sclerosis of valve leaflets. Severe calcification causes deformation of the valve tissue and ultimately aortic stenosis (AS). It is the most common form of valve disease in the Western world and it will become an increasing health burden with an ageing populations (Stewart et al., 1997; Nkomo et al., 2006; Takkenberg et al., 2008; Osnabrugge et al., 2013).

Calcification of the aortic valve is an active, proliferative process that results from inflammation, fibrosis and bone matrix formation (Rajamannan et al., 2011; Mathieu et al., 2014; Rutkovskiy et al., 2017), mediated by the signaling pathways common to valvulogenesis and osteogenesis (Dutta and Lincoln, 2018). Currently, the main therapeutic option is open heart surgery and replacement with a valve prosthesis, although endovascular or minimally invasive techniques are being introduced for an increasing number of patients. Considering the costs and risks involved in surgical and endovascular replacement, pharmacological inhibition of CAVD ought to be a priority of research (Bhatia et al., 2016).

The basic mechanisms of aortic valve calcification are still poorly understood. Unraveling the cellular and molecular mechanisms of valve calcification may open up new therapeutic options, which is why researchers are in need of reliable and relevant models (Pawade et al., 2015). The valve interstitial cells (VIC) are believed to play a key role in the calcification process and VIC in culture appear to be the most relevant *in vitro* model for valve calcification (Takkenberg et al., 2008; Mathieu et al., 2014; Rutkovskiy et al., 2017). This is particularly true due to the lack of good animal models–the pathophysiology of aortic stenosis is quite unique to humans (Sider et al., 2011; Gomez Stallons et al., 2016). Experimentally induced calcification in VIC is an accurate and affordable model of *in vitro* aortic valve calcification. It is also suitable for screening potential pharmacological inhibitors. Different techniques or variants of *in vitro* models that are used in investigations leading to inconsistencies across the studies (Goto et al., 2019). This is also true for *ex vivo* and *in vivo* models of the disease, where the methods vary wildly.

Presently available models to study aortic valve calcification are not optimal, however, they are what we have for now. Exact techniques for measuring the amount of calcification as well as exact content of trace elements are important parts of research studies. Good methods for evaluating aortic valve calcification by different imaging techniques have become increasingly important due to catheter-based implantation of valve prostheses. Exact information about the calcification of the valve and valvular annulus are decisive for a successful result. The purpose of this article is to give an overview of methods for investigating cellular and molecular mechanisms of aortic valve calcification as well as techniques to measure the amount of calcium and calcification. Cultures of VIC have been given special attention as they are extensively used to study the cellular and molecular mechanisms of calcification. Furthermore, we present an overview of different *in vitro*, *ex vivo*, and *in vivo* models to study calcification as well as methods to investigate calcification and CAVD in patients.

## 2 Morphology of the Aortic Valve

Human aortic valve leaflets have three main layers: the *fibrosa* facing the aorta, the *spongiosa* in the middle and the *ventricularis* facing the ventricle. This tri-layer structure is populated with VIC, and the surface is covered with a monolayer of valve endothelial cells (VEC). VICs are able to synthesize and regulate remodeling of extracellular matrix components. VEC function as a barrier, a signaling interface, they produce a number of substances that potentially regulate VIC, and may have an active role in valve calcification (Tao et al., 2012; Rattazzi and Pauletto, 2015). Injury to the valve endothelium might even be the trigger of the whole process. In a healthy valve, VIC are quiescent and have characteristics akin to fibroblasts. Under certain conditions VIC can undergo differentiation to either myofibroblasts or osteoblast-like cells (Rutkovskiy et al., 2017). At the time of surgery, approximately 13% of stenotic valves have inclusions of osteoblasts and osteoclasts along with organized lamellar bone matrix. Around 83% have signs of dystrophic calcification, possibly mediated by myofibroblasts (Mohler et al., 2001). While the exact mechanism is unclear, the myofibroblasts may contract the extracellular matrix, creating cellular aggregates (nodules), where the cells undergo apoptosis. This leads to calcium phosphate precipitation around apoptotic bodies (Chen and Simmons, 2011) and turns micro-calcification into macro-calcification.

## 3 Measuring Amounts of Calcium

### 3.1 In Cell Cultures

#### 3.1.1 Alizarin Red Staining and Quantification of Calcification in Cell Cultures

Alizarin Red (1.2-dihydroxyanthraquinone) staining is the most common method used to assess calcification in cell culture. It stains mineralized matrix, binding to different bivalent ions, mostly calcium. Although it is not the most accurate method to detect calcium content, it is optimal in terms of time consumption, simplicity, and cost (Bowler and Merryman, 2015). Alizarin Red provides a visual picture of calcium distribution in cell culture. It is also possible to quantify the signal by extracting the dye with acetic acid and measuring its concentration spectroscopically. Another common method for identification of calcium deposits is von Kossa staining. The latter dye unlike Alizarin Red reacts with phosphates and carbonates in calcium deposits (Puchtler and Meloan, 1978; Prins et al., 2014). An alternative method for quantifying calcification is cetylpyridinium chloride extraction. This method is less labor intensive, but less sensitive than Alizarin Red (Gregory et al., 2004). Another method to quantify calcium in cell cultures is the colorimetric method. This method needs solubilization of calcium deposits with HCl. The calcium content of HCl supernatants is then determined colorimetrically using commerical kits. This technique can also be used for tissue biopsies (Jono et al., 2000; Poggio et al., 2014; Hortells et al., 2015; Gayrard et al., 2020). In addition to that, many other calcium deposits detection methods have been developed including fluorescent and peptide−based dyes (Lee et al., 2012; Macri-Pellizzeri et al., 2018; Sim et al., 2018).

### 3.2 *Ex vivo*

#### 3.2.1 Microscopy

Standard light microscopy today has a limited place in the analysis of mineral content. However, some information may be obtained with polarized light microscopy. For instance, ectopic deposits and amorphous masses may be characterized as containing apatite (Cottignoli et al., 2015a). More detailed information about mineral content can be detected by confocal microscopy collecting Raman spectra. This has been used in a few studies attempting to characterize the mineral content of calcified valves, sometimes in combination with infrared spectra (Mangialardo et al., 2012) or powder X-ray diffraction (Gourgas et al., 2020). Raman spectra can assess the crystallinity of mineral deposits, and peaks in the spectra suggest that the main mineral in calcified valves is carbonated hydroxyapatite (Gourgas et al., 2020). Unfortunately, however, there are severe limitations as to how much qualitative and quantitative information can be obtained regarding mineral content and composition of calcified aortic valves using techniques based on light microscopy.

#### 3.2.2 Electron Microscopy

Both scanning and transmission electron microscopy have been used in several studies to characterize the biomineralization of calcified aortic valves and in particular their morphology (Mangialardo et al., 2012; Danilchenko et al., 2013; Cottignoli et al., 2015a; Cottignoli et al., 2015b; Gourgas et al., 2020). These techniques also show disturbances in the organic parts of the leaflets and the extracellular matrix such as disorganized bundles of collagen fibers. It is also described “the presence of biological niches within the calcified extracellular matrix, small, unfilled cavities inside rock that may be formed through a variety of processes” (Cottignoli et al., 2015b). These techniques also show details of different shapes of the crystalline structure: semispherical, laminar crystals, and spherical particles that make the calcified masses. The masses are described as bioapatite and also form needle or rod like crystals (Cottignoli et al., 2015b). Electron microscopy is the method with the highest resolution, but it is very labor-intensive, making its routine application difficult. However, additional methods are necessary for qualitative studies of calcification and crystals.

#### 3.2.3 Micro-Computed Tomography (Micro-CT)

A novel method to describe the morphology and density of calcification and minerals in explanted aortic valves is micro-CT (Orzechowska et al., 2014). Micro-CT has a resolution of one micron; it is suitable for studying porosity, bone thickness, density, particle size, fiber orientation, etc. The level of x−ray signal attenuation is proportional to the material density and thickness. This could be interpreted as different levels of calcification. Soft tissue presents with very low attenuation calcifications, while bone matrix causes high attenuation, which creates high contrast images. Using micro-CT it is possible to assess the amount of calcified tissue in relation to the total volume of valve leaflet or the whole valve. In a study of explanted aortic valves, micro-CT showed a strong correlation between the amount of calcification and the severity of aortic stenosis (Chitsaz et al., 2012). Another study using micro-CT identified aortic valve deposits as B-type carbonate-containing hydroxyapatite (Orzechowska et al., 2014). In general, however, this technique does not give detailed information on the mineral composition, rather information on material density. Thus, it may give ratios between soft tissue and more calcified (harder) tissue.

#### 3.2.4 Inductively Coupled Plasma Optical Emission Spectrometry and Inductively Coupled Plasma Mass Spectrometry (ICP-OES and ICP-MS)

The information obtained from this technique (chemical analysis) is not comparable to the others, as the approaches described above are able to study the crystal morphology, particle size, etc. However, ICP-OES and ICP-MS are techniques used for elemental analysis concentration (Hanć et al., 2011). There are some drawbacks, such as, it is a destructive technique: you have to digest the samples before analysis.

These techniques are superior for quantifying calcium (see 4.2.2.) and a broad series of trace elements (Heitkemper et al., 1994; Baralkiewicz et al., 2007; Sneddon and Vincent, 2008; Kreitals and Watling, 2014; Paraskova et al., 2015). In particular, these techniques have been quite extensively used for industrial purposes with ramifications for biology and forensic medicine (Carpenter, 1985). In veterinary medicine this technique has been used, to measure 14 trace elements from bovine liver biopsies (Braselton et al., 1997). Recently, there has been a shift towards the utilization of ICP-MS which offers a low detection limit combined with high sample throughput. ICP-MS also offers analysis of at least 25 trace elements in a biological sample. With a few exceptions, the lower detection limit of trace element and minerals is 1 nmol/L or less (Wilschefski and Baxter, 2019).

#### 3.2.5 Miscellaneous, Minerals and Crystals of Calcified Aortic Valves

Among other methods used for characterization of biomineralogy and chemical composition are X-ray microanalysis coupled with energy-dispersive X-ray (Danilchenko et al., 2013) and direct chemical analysis, X-ray diffraction and Fourier transform infrared (Prieto et al., 2011). However, these techniques are usually used as adjunctive methods and in combinations with other techniques. Until quite recently, there was little knowledge regarding the analysis of the exact composition of calcified aortic valves. However, modern techniques have taught us more about the composition, structure, and formation of calcified valves. Mineralogical analyses of calcified valves to gain information about crystallization may be important and possibly an underestimated part of understanding the calcification process.

### 3.3 Imaging *in vivo*

Reliable imaging of the aortic valve has become increasingly important in recent years in parallel with the increase of catheter-based valve replacements (Bettinger et al., 2017; Francone et al., 2020; Mittal and Marcus, 2021). Usually a multimodality approach is recommended for evaluation of calcification, the characteristics of the valve itself and the aortic root. There are excellent reviews discussing imaging far beyond the scope of this overview (Salemi and Worku, 2017; Pawade et al., 2019; Francone et al., 2020; Ternacle and Clavel, 2020; Tzolos et al., 2020; Fletcher et al., 2021).

#### 3.3.1 Echocardiography

Echocardiography is the standard clinical basis of all heart valve evaluations. It is safe, not expensive, widely available, and non-invasive. The key assessment criteria of aortic stenosis are combination of aortic valve area, mean gradient across the valve, and peak flow velocity. Details of the method including its limitations are beyond the scope of this review (Chong et al., 2019). Important to note is that echocardiography cannot quantify calcium*.* However, echocardiography is the standard technique to evaluate valve function, degeneration, leaflet stiffness due to fibrosis, and calcification of the aortic valve with the development of aortic stenosis. Transesophageal echocardiography provides better imaging than transthoracic, in particular to differentiate between tri- and bicuspid aortic valves (Yousry et al., 2012; Yousry et al., 2015).

#### 3.3.2 Computed Tomography (CT)

CT is the method to choose to evaluate and quantify aortic valve calcification as calcium score measured by Agatston score “which accounts for both the density and volume of CT-measured calcium and correlates closely with the weight of calcium in explanted aortic valves” (Kang et al., 2010). Aortic valve calcium score also correlates well with calcific aortic valve disease progression and prognosis (Messika-Zeitoun et al., 2007; Nguyen et al., 2015) and is closely associated with severity of aortic stenosis measure by echocardiography (Cowell et al., 2003; Cueff et al., 2011; Tastet et al., 2017). For exact evaluation of the role of assessment of aortic valve calcification by CT, it is necessary to be aware of a series of pitfalls as described by *Pawade et al.* (Pawade et al., 2019). This is particularly important in younger patients (<51 years) with bicuspid aortic valves where *Shen et al.* found no correlation between mean gradient across the aortic valve and aortic valve calcium density (Shen et al., 2017). Several studies have also shown that women have lower calcification loads than men for the same aortic stenosis severity (Aggarwal et al., 2013; Gourgas et al., 2020). Standard CT cannot detect the early stages with micro-calcification, it can only visualize confluent areas of macro-calcification (Bailey et al., 2016). However, recently contrast-enhanced CT has been shown to be able to assess not only calcium, but also non-calcific (fibrotic) aortic valve composition, allowing assessment of early CAVD (Cartlidge et al., 2021; Grodecki et al., 2021).

#### 3.3.3 Magnetic Resonance Imaging (MRI)

MRI is not routinely used and is far less widespread than CT. It is rather a supplementary imaging technique. However, it has an increasing role in the planning of endovascular aortic valve procedures (Mittal and Marcus, 2021). In the recent consensus document by the European Society of Cardiovascular Radiology, it is explicitly stated that MRI have many potential advantages in such situations (Francone et al., 2020). This includes all necessary measurements of the valve and the aortic root as well as evaluation of ventricular function and the aorta. Furthermore, MRI has some distinct advantages to avoid the use of contrast in cases with severe kidney failure. Unfortunately, it does not provide reliable calcium score.

#### 3.3.4 Positron Emission Tomography (PET)

PET uses radioactive isotopes, which concentrate in regions with high metabolic activity, thus being able to detect changes on the molecular and cellular level before anatomic changes occur. 18F-sodiumfluoride accumulation was found to correlate with calcification in the aortic valve (Dweck et al., 2014) and it is able to detect micro-calcification in the vasculature (Vancheri et al., 2019) and in valves (Hutcheson et al., 2014). 18F-sodiumfluoride binds to hydroxyapatite on calcified nodules and is quantitatively shown to be associated with faster progression of CAVD (Dweck et al., 2014; Jenkins et al., 2015). Furthermore, accumulation of 18F-sodiumfluoride is also associated with bio-prosthetic aortic valve degeneration (Cartlidge et al., 2019). So far PET is primarily a research tool and less used in clinical investigations, partly due to costs and lower availability in clinical practice. However, PET may be helpful to develop our understanding of aortic stenosis, both its molecular background as well as its development, risk stratification, and progression in patients (Pai et al., 2006; Rojulpote et al., 2020). In particular, it may be a powerful tool when it localizes the process in 3D, in combination with CT and/or MRI (Tzolos et al., 2020).

## 4 Collection of Human Aortic Valves for Cell Isolation

For studies in cell cultures, cells from human aortic valves are preferred in order to eliminate species differences. Exceptions are relevant when *in vivo* animal experiments are performed or when using transgenic models. Calcified human aortic valves are fairly easy to obtain if the laboratory is situated in the proximity of a cardiac surgery unit. Calcified aortic valves can be harvested from patients with aortic valve stenosis undergoing aortic valve replacement. Cells from a valve can be freshly isolated and usually retain moderate to high degree of viability. The degree of cell viability/quantity/proliferation for each donor is individual. It depends among others on the level of the valve calcification where severe calcified valves result in the inferior cell isolation yield. Of note: patients with rheumatic aortic valve stenosis, a late inflammatory complication of group A *Streptococcal pharyngitis*, represent a totally different disease (Wallby et al., 2013) which is not included or discussed here.

Healthy valves are less readily available. There are several potential sources of non-calcified human aortic valves (Rutkovskiy et al., 2017). The ideal one is from donor hearts that were considered unsuitable for transplantation. Other possibilities include valves from explanted hearts of heart transplant recipients.

For isolation of cells from explanted aortic valves, timing is critical, in particular for the isolation of VEC. By placing the leaflets in saline immediately after excision and keeping at +4 C enhances viability, opening a window of several hours for VEC isolation. VIC are less sensitive to time before isolation, however, we recommend isolation of VIC within the first 24 h. According to our experience with aortic valve cells from autopsy material acceptable viability can be expected for up to 24 h post mortem which is in accordance of what has been reported earlier (Gall et al., 1998). Gender may also be important: valves from men have more advanced calcification at the same age as women, whereas stenotic valves from women have increased levels of fibrosis compared to men (Aggarwal et al., 2013). It is also important to avoid mixing bicuspid (BAV) and tricuspid (TAV) aortic valves since there are differences in the molecular and cellular mechanisms that underlie calcification of BAV and TAV (Kostina et al., 2018).

## 5 Endothelial and Interstitial Cells From Human Aortic Valves

Handling of cells and cell cultures are presented in more details than other techniques here because it is probably the most widely spread and concise method to study the basic cellular and molecular events of aortic valve calcification. Additionally, cell culture models are also used for initial screening of potential inhibitory drugs (Dutta et al., 2021; Natorska et al., 2021; Parra-Izquierdo et al., 2021; Wang et al., 2021).

### 5.1 Isolation and Culture

**FIGURE 1:**
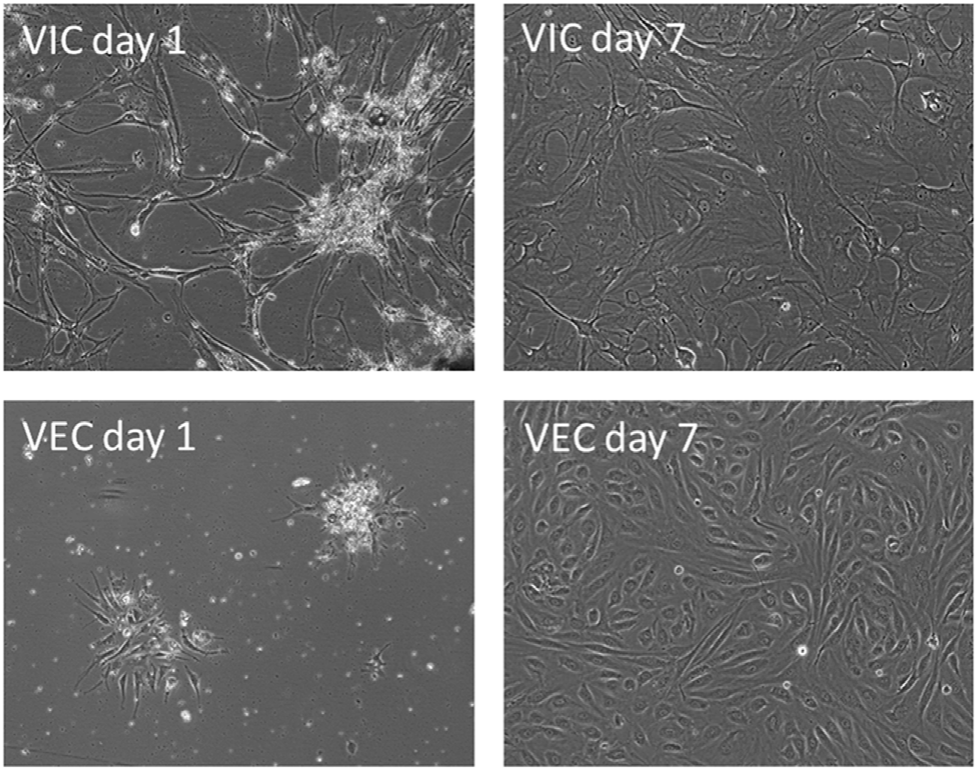
Interstitial (VIC) and endothelial (VEC) cells isolated from human aortic valves shown on first and seventh days after isolation. Phase contrast microscopy.

The most widely used and reproducible techniques for isolation of VEC and VIC have been derived from methods that originally employed porcine material (Gould and Butcher, 2010). The quality of plastic on which the cell culture is seeded is crucial for good VIC growth. Normal practice is to use standard cell growth medium containing DMEM supplemented with 10–15% fetal bovine serum (FBS) and antibiotics. The day after isolation, the VICs are usually visible, and they present a fibroblast-like morphology (Figure 1). During cultivation, the media is changed twice a week until a confluence of 70–80% is attained (usually within 1 week, see Figure 1). At this point the VIC are harvested using trypsin/EDTA and seeded at a high density (we recommend to not exceed ratio of 1:2), which is a crucial factor for survival of VICs isolated from calcified valves. It is recommended to passage VICs after achieving density around 90–95%. VECs are usually isolated by swabbing cells from the surface of the leaflet or via vortexing collagenase−treated leaflets. On the second day in culture, the rosette-like colonies of VECs should form. VECs are grown until a confluence of 70–80% is attained (usually within 1 week, Figure 1). VEC then passaged at a ratio of 1:3. Primary cells in culture are known to change particular properties with each passage, thus the number of passages is important to report (Yperman et al., 2004; Goto et al., 2019). An important precaution with regards to the culture of primary VECs and VICs is to ensure they are mycoplasma-free, therefore all cultures should be kept in a quarantine area until a *mycoplasma* test has been conducted.

### 5.2 Purification and Characterization

**FIGURE 2:**
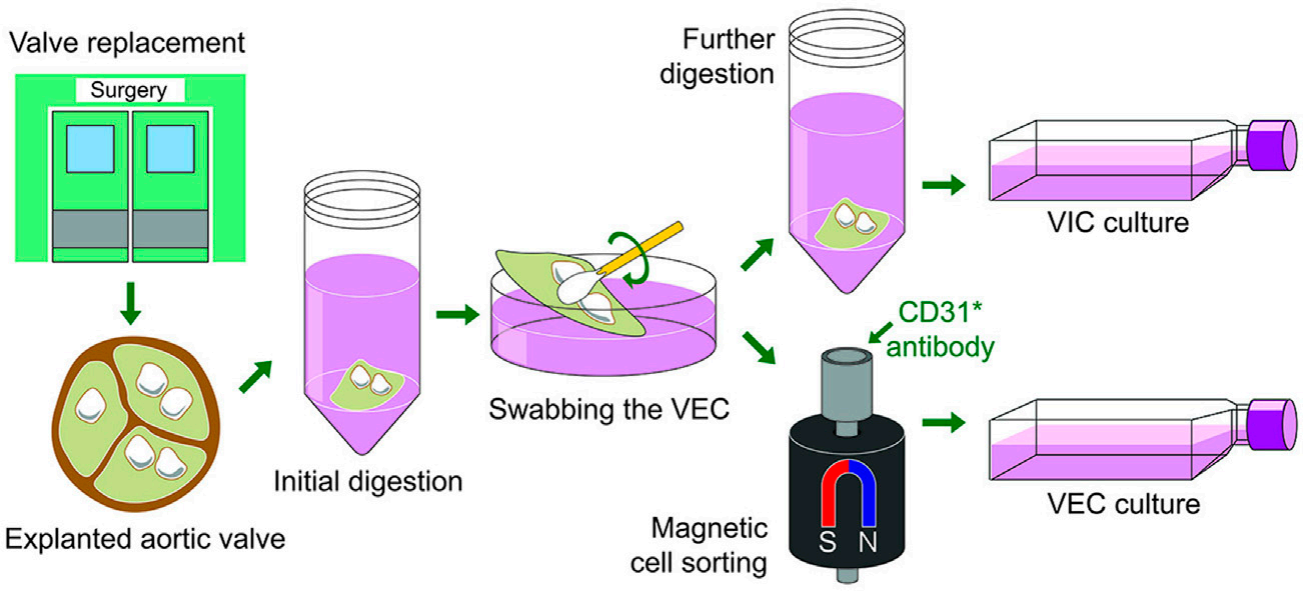
A schematic overview of the main steps of the calcified human aortic valves collection, digestion and valvular interstitial and endothelial cells isolation.

**FIGURE 3:**
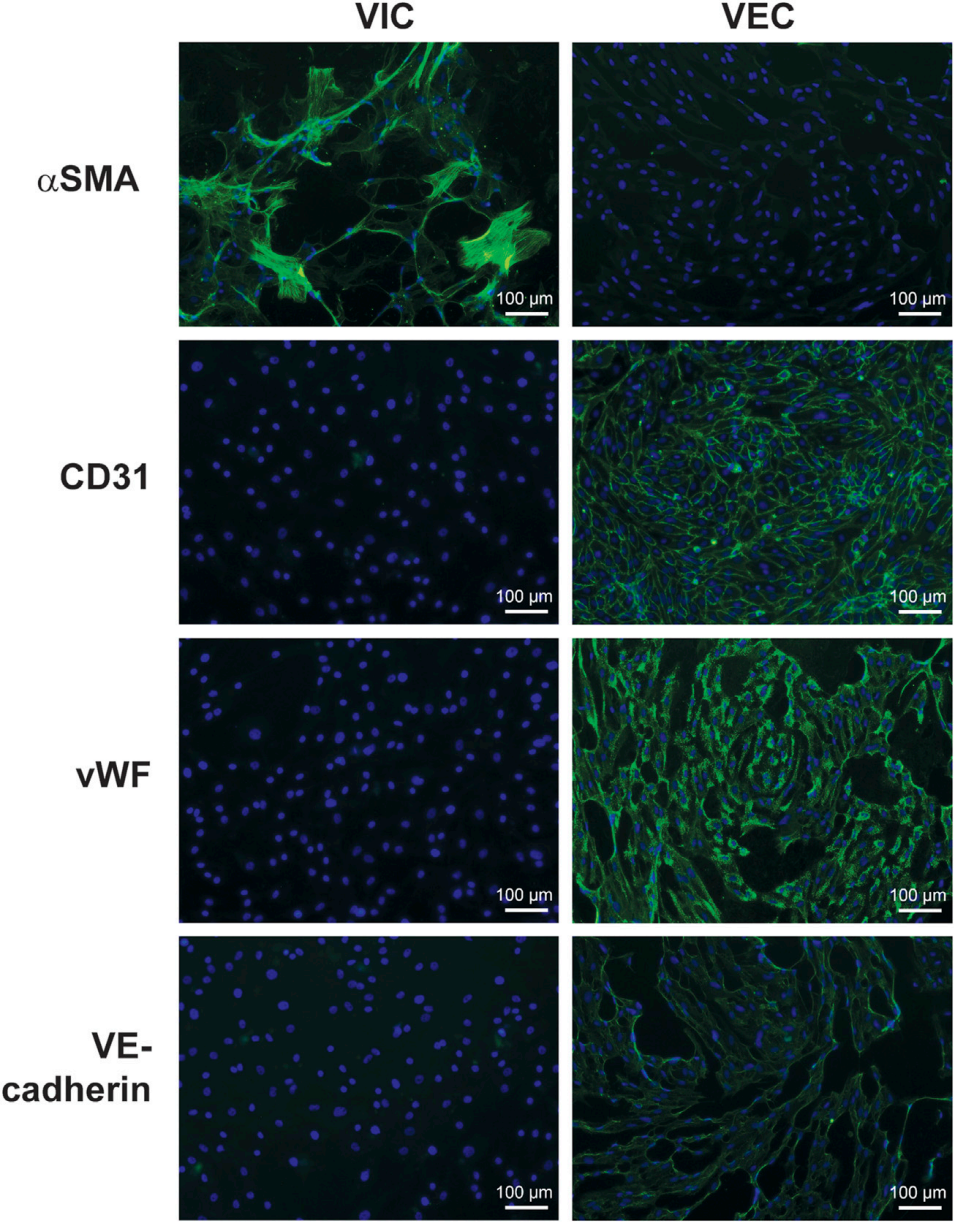
Immunofluorescence staining for fibroblastic and endothelial markers in valve interstitial (VIC) and endothelial cells (VEC). Cells were isolated from human aortic valves with calcification (n = 3) and separated by magnetic-activated cell sorting. The pictures show expression of alpha-smooth muscle actin (αSMA) in VIC and cluster of differentiation 31 (CD31), vascular endothelial cadherin (VE-Cadherin), and von Willebrand factor (vWF) in VEC. The nuclei were stained with Hoechst 3342 (blue).

**FIGURE 4:**
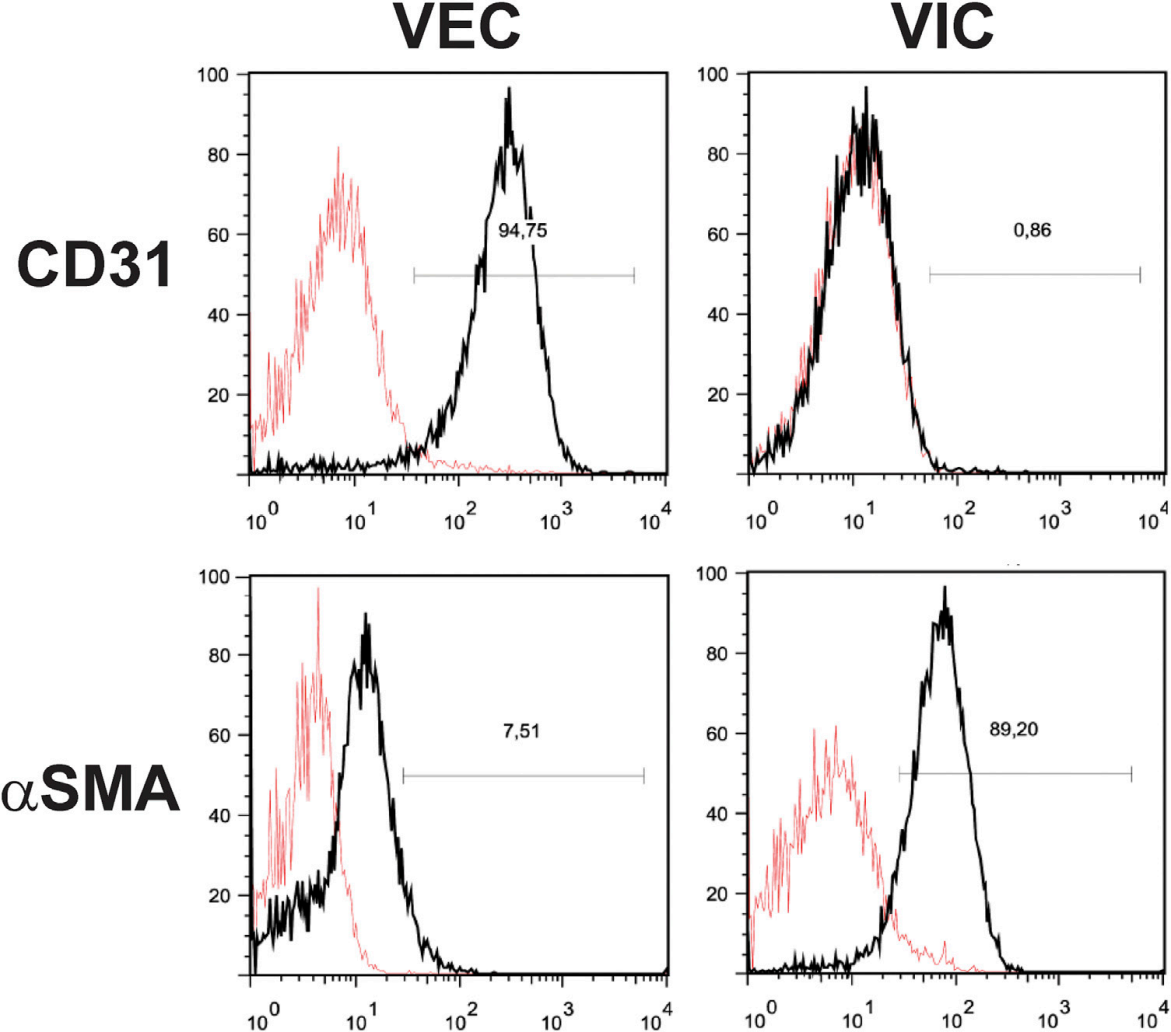
Fluorescence-activated cell sorting (FACS) analysis of valve endothelial (VEC) and interstitial cells (VIC). Cells were isolated from aortic valves with calcification (n = 3). A representative image shows expression of the typical fibroblastic marker protein alpha-smooth muscle actin (αSMA) in VIC, but not in VEC and expression of typical endothelial cell marker CD31 in VEC, but not in VIC.

Isolated VEC from calcified valves will invariably be contaminated with VICs, which will threaten to outgrow them over time. Therefore, an enrichment step is highly recommended, for instance, magnetic-activated cell sorting 1 week after initial isolation (Gould and Butcher, 2010). Human VEC can be enriched using the surface markers PECAM-1/CD31 (Platelet endothelial cell adhesion molecule-1), providing a discriminatory marker for enrichment. The main steps or the cell isolation procedure are summarized in Figure 2. Following enrichment, it is recommended to assess the purity of the respective cell populations using markers that allow the delineation of each cell type. The VEC population may be assessed using flow cytometry against the endothelial marker CD31 (Gould and Butcher, 2010) and/or von Willebrand factor (vWF) (Gould and Butcher, 2010) and VE-cadherin (Farrar and Butcher, 2014) (Figure 3). We routinely observe high purity, with 95% of the population being CD31 positive (Figure 3). Flow cytometry data are well in line with the immunocytochemistry staining. The cell population should not exhibit alpha-smooth muscle actin expression (αSMA) in immunocytochemistry assessment (Figure 3). However, using flow cytometry we observed that approximately 7.5% cells are positive for αSMA. This can suggest either VIC culures are contaminated or the presence of cells bearing both markers, such as VEC undergoing mesenchymal transition (Figure 4).

αSMA is common marker used to separate human VIC population from VECs (Gould and Butcher, 2010) and is useful to assess the purity of the VIC population. The expression of αSMA is low in healthy human valves, and relatively high in calcified valves (Olsson et al., 1994). Higher expression of αSMA is associated with myofibroblastic differentiation, which is one of the hallmarks of CAVD. It is suggested that cultured VICs can spontaneously differentiate into myofibroblast-like cells as they increase αSMA expression with time (Pho et al., 2008; Monzack and Masters, 2011; Latif et al., 2015a; Porras et al., 2017). The spontaneous myofibroblast differentiation is believed to be a result of the novel physical environment, which influences cells via mechanoreceptors. It is well established that rigid substrates promote myofibroblast differentiation in fibroblasts. It may be related to the physical properties of the substrate (culture plastic) as stiffer substrates are known to promote myofibroblast phenotype (Yip et al., 2009). Some authors propose culturing VIC in “fibroblast medium” to potentially reduce expression of myofibroblastic markers such as αSMA, transgelin, and extra domain-A fibronectin (Latif et al., 2015a; Porras et al., 2017). Alternatively, one can try to use cells with low passage numbers to be as close to the original phenotype as possible.

Using flow cytometry, we observed that approximately 90% of VIC from calcified aortic valves were positive for αSMA (Figure 4). The presence of αSMA was further verified by immunostaining (Figure 3). Possible contamination with VEC in the VIC population was assessed by immunostaining for vWF, CD31, and VE-cadherin (Figure 3). This can be further validated using flow cytometry for CD31 (Figure 4). There has been efforts to find additional markers that are highly expressed in human VICs, for example, vimentin (Latif et al., 2007; Latif et al., 2015a), prolyl-4-hydroxilase (Taylor et al., 2000; Osman et al., 2006a) and markers of bone-morrow mesenchymal stem cells: fibroblast surface antigen (CD90) (Latif et al., 2007; Latif et al., 2015a) and CD44 (Latif et al., 2007). The relevance of these markers for separation of human VICs from VEC have not been confirmed. We observed expression of vimentin and CD90 in both human VEC and VIC populations and do not recommend the use of these markers for purification of human VIC. It has also been suggested that calponin can be utilized as a VIC marker, especially associated with progression of CAVD (Plazyo et al., 2018; Bogdanova et al., 2019).

### 5.3 2D or 3D Culture Models of Human Aortic Valve Cells

#### 5.3.1 Interstitial Cells

2D models of culturing VIC and VEC are most common, although 3D cultures are used as well. 3D cultures have been suggested to have some advantages providing a more physiologically relevant model for the cells because VIC are influenced by their microenvironment (Hjortnaes et al., 2015; Ravi et al., 2015; Hjortnaes et al., 2016; van der Valk et al., 2018; Bracco Gartner et al., 2019). This is highlighted by Hjortnaes and co-workers when cell are cultured in 3D hybrid hydrogels composed of hyaluronic acid and gelatin (Hanć et al., 2011). They state the following: “The elastic modulus of 3D hydrogels used in our study (∼20kPA) corresponds to the perceived modulus of the fibrosa as measured by micropipette aspiration up to 21 kPA” Furthermore, “We previously showed that the 3D hydrogel platform maintains a quiescent VIC phenotype identified in healthy heart valves, thus providing a platform to study phenotypic changes associated with CAVD” as well as “The 3D approach presented in this work can maintain healthy quiescent VIC population and thus can model the entire cellular process”. However, there are several limitations of the 3D model. The hydrogel platform is static and the composition of their hydrogel is different when compared to the *in vivo* extracellular matrix. The 3D cultures are more difficult to subject to mechanical stimulation and the diffusion through the gel should be taken into account when performing chemical stimulations. Although 3D platform may in some ways be attractive, 2D cultures for VIC are still leading in the field due to simplicity and standardization. For general use, the superiority of 3D cultures use can so far be discussed.

#### 5.3.2 Co-Cultures of Valve Endothelial and Interstitial Cell

Recent studies of the molecular and cellular mechanisms of CAVD have emphasized the importance of VEC-VIC interactions. Porcine VIC have reduced expression of the myofibroblastic gene αSMA when co-cultured with VECs (Butcher and Nerem, 2006), implying that VECs are involved in the regulation and maintenance of the VIC phenotype. This is also corroborated by several studies demonstrating that VECs inhibited myofibroblastic or osteogenic differentiation of porcine VIC in co-culture (Kennedy et al., 2009; Richards et al., 2013; Gould et al., 2014), suggesting an important role of VEC-VIC interaction for cellular valve homeostasis. Dysfunction or denudation of VECs, have also been implicated as an initiator of VIC transformation leading to calcification (Leopold, 2012; Gomel et al., 2018; Hulin et al., 2018). It was recently suggested that VEC isolated from different sides of the valve have a different effect on the VIC calcification through cadherin-11 (Johnson and Merryman, 2021).

Static 3D co-cultures of human VEC and VIC are suitable for studies of cell type interactions. We seeded VIC pre-mixed with collagen and when the gels were cast, VEC were seeded on top. The endpoints included gene expression changes, as well as the contraction of collagen gels by the VIC, being a function of their myofibroblast differentiation. More details on this topic are provided in Section 5.4.3. We also have positive experience with 2D co-cultures where the VIC are seeded at 90% density and then VEC are seeded directly on top of the VIC in the amount that is sufficient to achieve the same density in the top monolayer.

An interesting version of 3D co-cultures was recently reported by van der Valk et al., where they engineered a 3D-bioprinted model of a human aortic valve (van der Valk et al., 2018). In this study the aortic leaflet tissue was mechanically tested after micro-dissection of different layers. Leaflets were then constructed by bioprinting of 3D hydrogels with encapsulated human VIC. The hydrogels had been tuned to duplicate specific mechanical characteristics of the leaflets. It is too early to conclude how helpful this model is due to limited usage data.

### 5.4 Osteogenic Differentiation of Valve Interstitial Cells

Osteoblast- and osteoclast-like cells have been identified histologically in human calcified aortic valves (Mohler et al., 2001), but not in healthy aortic valves. Many markers that are attributed to osteoblasts have been found in valves of patients with CAVD and the majority of these markers are also expressed by VIC differentiated into osteoblast-like cells *in vitro* (Osman et al., 2007; Galeone et al., 2013; Zhang et al., 2014). The most common formulation of osteogenic medium that triggers calcification and expression of osteogenic markers in human VICs include beta-glycerophosphate, dexamethasone and ascorbic acid, which can be substituted with vitamin D (Osman et al., 2006a; Osman et al., 2006b; Osman et al., 2007; Babu et al., 2008; Galeone et al., 2013). Beta-glycerophosphate is the most potent component of most osteogenic media, as it donates a phosphate group to calcium ions to form calcium phosphate crystals, the main ingredient in mineral bone matrix. Beta-glycerophosphate induces transdifferentiation into osteoblast-like cells, thus increasing osteoblast activity and subsequent calcification (Babu et al., 2008). Dexamethasone stimulates both osteogenic and adipogenic differentiation depending on its concentration. The typical concentration that induces osteogenic differentiation is 0.1 µM, whereas higher concentrations are used to induce adipogenic differentiation (Zhao et al., 2018). Ascorbic acid is an additional cofactor that facilitates osteogenic differentiation by increasing collagen I synthesis (Ishikawa et al., 2004) and secretion (Langenbach and Handschel, 2013). The length of treatment in the majority of studies is 21 days (Osman et al., 2006a; Osman et al., 2006b; Osman et al., 2007; Babu et al., 2008). Basic osteogenic medium can be supplemented with BMP2, which has been demonstrated to be important for valve calcification (Zhang et al., 2014). It has been shown that treatment of human VIC with ATP (Osman et al., 2006a); BMP2 (Bone morphogenetic protein 2), BMP4, BMP7, TGFβ-1 or TGFβ-3 (Osman et al., 2006b) for 21 days can activate expression of alkaline phosphatase (*ALP*), which is a marker of late-stage osteoblastic differentiation. In our hands, the strongest effect was obtained when VICs were stimulated for 21 days with a basic osteogenic medium containing standard cell growth medium (DMEM, 10% FBS) supplemented with 10 mM beta-glycerophosphate, 0.1 µM dexamethasone and 50 µM ascorbic acid. This regimen induced reproducible and robust calcification (Bogdanova et al., 2019).

Another popular formulation of medium that promotes osteogenic differentiation of human VIC (termed “pro-calcifying medium”) include DMEM supplemented with 5% FBS, 2 mM NaH_2_PO_4_ and 50 μg/ml ascorbic acid (Bouchareb et al., 2015; Rogers et al., 2017; Schlotter et al., 2018). Gotto *et al.* (Goto et al., 2019) showed that the calcification potential of human VIC decreased with passage number in osteogenic medium, but not in pro-calcifying medium. Passage-dependent calcification of VIC cultured in osteogenic medium is regulated by abundance of tissue non-specific alkaline phosphatase (TNAP), an enzyme that hydrolyzes β-glycerophosphate to inorganic phosphate, which can be incorporated into calcium phosphate crystals promoting calcification. TNAP also plays a key role in mineralization by degrading inorganic pyrophosphate (calcification inhibitor) and providing free inorganic phosphate to induce calcification (Hui and Tenenbaum, 1998). Pro-calcifying medium contains inorganic phosphate and therefore does not require TNAP for the calcification process in VIC (Goto et al., 2019). Proteomic analysis of human VICs revealed induced expression of fibrosis- and calcification-related proteins under treatment with both osteogenic and pro-calcifying medium compared to control cells without stimulation, some of these proteins were shared between the two treatment groups (Schlotter et al., 2018). Further studies are required to gain a better understanding of which of the culture media best reflect the natural conditions of aortic valve calcification.

#### 5.4.1 Myofibroblastic Differentiation of Valve Interstitial Cells

Myofibroblasts are defined as fibroblasts that have some properties of smooth muscle cells and are characterized by the presence of stress fibers composed mainly of αSMA, providing the ability to contract the extracellular matrix (Tomasek et al., 2002). The myofibroblast-like cells play an important role in extracellular matrix remodeling in the pathogenesis of aortic valve calcification (Liu et al., 2007). High expression of αSMA is a well described marker of myofibroblasts (Tomasek et al., 2002) which is increased in calcified aortic valves (Olsson et al., 1994). A recent study has proposed that MAPK/ERK as a potential pathway involved in myofibroblast calcification in CAVD (Gonzalez Rodriguez et al., 2021). In addition to αSMA, Calponin and SM22 (Transgelin) are established markers to identify myofibroblast-like cells in human (Latif et al., 2015b; Porras et al., 2017; Kostina et al., 2018).

TGFβ-1 (Transforming growth factor beta 1) is highly expressed in diseased aortic valve leaflets and has been the most extensively studied cytokine in relation to VIC activation and aortic valve calcification (Jian et al., 2003; Walker et al., 2004; Merryman et al., 2007; Hutcheson et al., 2012). As stated above, the majority of animal VICs are positive for αSMA, and its expression varies with the degree of myofibroblastic differentiation. In calcified valves this phenotype is usually widespread, but even then TGFβ-1 added to cultures can further promote it and thereby enhance αSMA expression (Walker et al., 2004; Kennedy et al., 2009; Monzack et al., 2009; Chen et al., 2011; Quinlan and Billiar, 2012). There appears to be species differences with respect to timing of myofibroblastic differentiation in porcine (Cushing et al., 2008) and ovine (Jian et al., 2002; Walker et al., 2004) VIC. After treatment with TGFβ-1 in low-serum medium, αSMA was detected after 24 h in ovine VIC (Gwanmesia et al., 2010), while αSMA was not detected until day 5 in porcine VIC (Gu and Masters, 2010). In our experience VIC isolated from both healthy and calcified human aortic valves have increased expression of αSMA and Calponin, analyzed by flow cytometry, after 4 days of stimulation with a myofibroblastic medium (DMEM, 1% FBS and 5 ng/ml TGFβ-1). Furthermore, stimulated cells from healthy valves are characterized by higher expression of these myofibroblastic markers indicating more prominent myofibroblastic differentiation in comparison with cells from calcified valves. In conclusion, a dynamic increase in αSMA and Calponin expression is a reliable myofibroblastic differentiation marker for human VIC isolated from healthy and calcified aortic valves.

#### 5.4.2 Role of Extracellular Matrix in Myofibroblastic Differentiation

**FIGURE 5:**
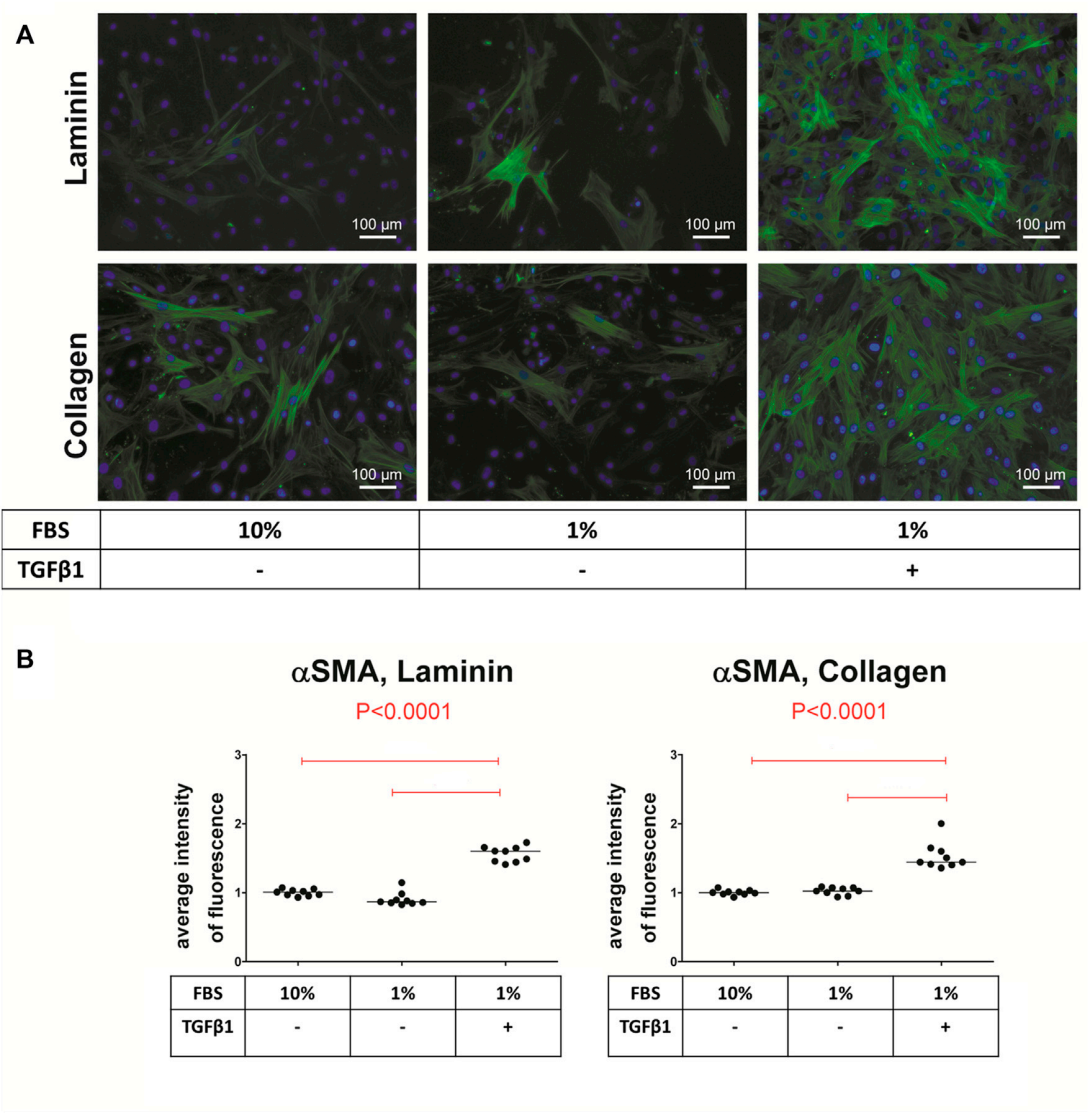
Immunofluorescence staining of valve interstitial cells (VIC) for alpha-smooth muscle actin (αSMA). **(A)** VIC were isolated from aortic valves with calcification (n = 9) and cultured for 14 days on either laminin or collagen, with 10% FBS without TGFβ-1, with 1% FBS without TGFβ-1 or with TGFβ-1. αSMA (green), cell nuclei (Hoechst 3342/blue). **(B)** Quantification of αSMA fluorescence, shown as scatter plot with median. Statistical differences were tested using ANOVA followed by Tukey test. Overall *p*-values from ANOVA analysis are shown in red.

The extracellular matrix plays a key role in the regulation of VIC phenotype and function, including the processes of differentiation (Gwanmesia et al., 2010). Moreover, it is speculated that TGFβ-1 may bind to components of the extracellular matrix and this interaction may be essential for its signaling (Chen and Simmons, 2011; Jenkins, 2008; Wipff and Hinz, 2008). Disruption of the extracellular matrix in valve leaflets in turn alters TGFβ-1 signaling in VIC, leading to remodeling and valve disease (Chen and Simmons, 2011; Jenkins, 2008; Wipff and Hinz, 2008). *In vitro* it has been demonstrated that different coatings on conventional tissue culture plates influence myofibroblastic differentiation in different ways (Chen and Simmons, 2011; Hinton and Yutzey, 2011). Collagen and laminin coatings increase both the calcification process and induction of αSMA in ovine VIC, whereas fibronectin has an opposite effect (Gwanmesia et al., 2010). Laminin, heparin, and fibrin, but not collagen or fibronectin promote nodule formation in porcine VIC (Rodriguez and Masters, 2009). Another factor that influences the differentiation of VIC into myofibroblasts is the rigidity of the matrix (mechanical properties). By varying the concentration of collagen in a 3-dimensional model, very different effects were observed: compliant matrices contribute to osteogenic differentiation and calcification, whereas stiff matrices promote myofibroblastic differentiation and calcification through apoptosis (Yip et al., 2009; Quinlan and Billiar, 2012; Wyss et al., 2012). In addition, the effect of TGFβ-1 on αSMA expression is proportional to the matrix stiffness (Chen et al., 2011). In 2D cultures, stiff substrates such as tissue culture plastic may be sufficient to promote VIC differentiation to myofibroblasts (Kennedy et al., 2009; Benton et al., 2008). A summary of coatings employed for myofibroblastic differentiation of animal VIC and their effect is shown in Table 1. Laminin and collagen are the most commonly used coating surfaces for culture of myofibroblasts (Monzack et al., 2009; Rodriguez and Masters, 2009; Yip et al., 2009; Gwanmesia et al., 2010; Chen et al., 2011; Quinlan and Billiar, 2012; Wyss et al., 2012). Figure 5 shows the comparison of αSMA expression in cells cultured either on laminin or collagen coating after stimulation with myofibroblastic medium.

**TABLE 1:** The effect of different coatings on myofibroblastic differentiation of cultured valve interstitial cells from different species.

Coating	Presence in Extracellular matrix of aortic valve	Model	Effect on myofibroblastic differentiation	References
laminin	in the basement membrane	sheep, porcine	↑	(Monzack et al., 2009; Rodriguez and Masters, 2009; Gwanmesia et al., 2010)
heparin	In spongiosa layer	porcine	↑	Rodriguez and Masters, (2009)
fibrin	found in valves of patients with aortic stenosis	porcine	↑	(Benton et al., 2008; Monzack et al., 2009; Rodriguez and Masters, 2009)
collagen	comprises a significant part of fibrosa layer	sheep, porcine	↑ (sheep model) ↑ (porcine model)	(Jian et al., 2002; Rodriguez and Masters, 2009; Gwanmesia et al., 2010)
fibronectin	in fibrosa layer	sheep, porcine	↓	(Benton et al., 2008; Rodriguez and Masters, 2009; Gwanmesia et al., 2010)
plastic	_	porcine	↑	(Benton et al., 2008; Kennedy et al., 2009; Monzack et al., 2009; Rodriguez and Masters, 2009; Gwanmesia et al., 2010)
PEG	_	porcine	↓	Benton et al. (2008)

#### 5.4.3 Myofibroblastic Contractility of Valve Interstitial Cells in 3D Cultures

Actin-myosin cytoskeleton of myofibroblasts is connected with components of extracellular matrix *via* cellular transmembrane receptors, the integrins, allowing cells to contract the surrounding extracellular matrix (Parizi et al., 2000). In order to provide relevant models that reflect the *in vivo* situation in humans with contraction inside the leaflets, VIC isolated from calcified valves can be incorporated into a 3D cell culture system, based on collagen gel (Bond et al., 2007). This allows measuring the contractility of VIC-derived myofibroblasts, which in turn demonstrates their functional attributes (Butcher and Nerem, 2004; Cushing et al., 2008). αSMA expression induced by TGF-β1 stimulation correlates with gel contraction confirming the contractile phenotype of VIC (Hinz et al., 2001). Blocking αSMA polymerization with cytochalasin D attenuates TGF-β1–induced contraction (Walker et al., 2004). These results confirm that VIC contract collagen gel due to their differentiation into myofibroblast-like cells.

**FIGURE 6:**
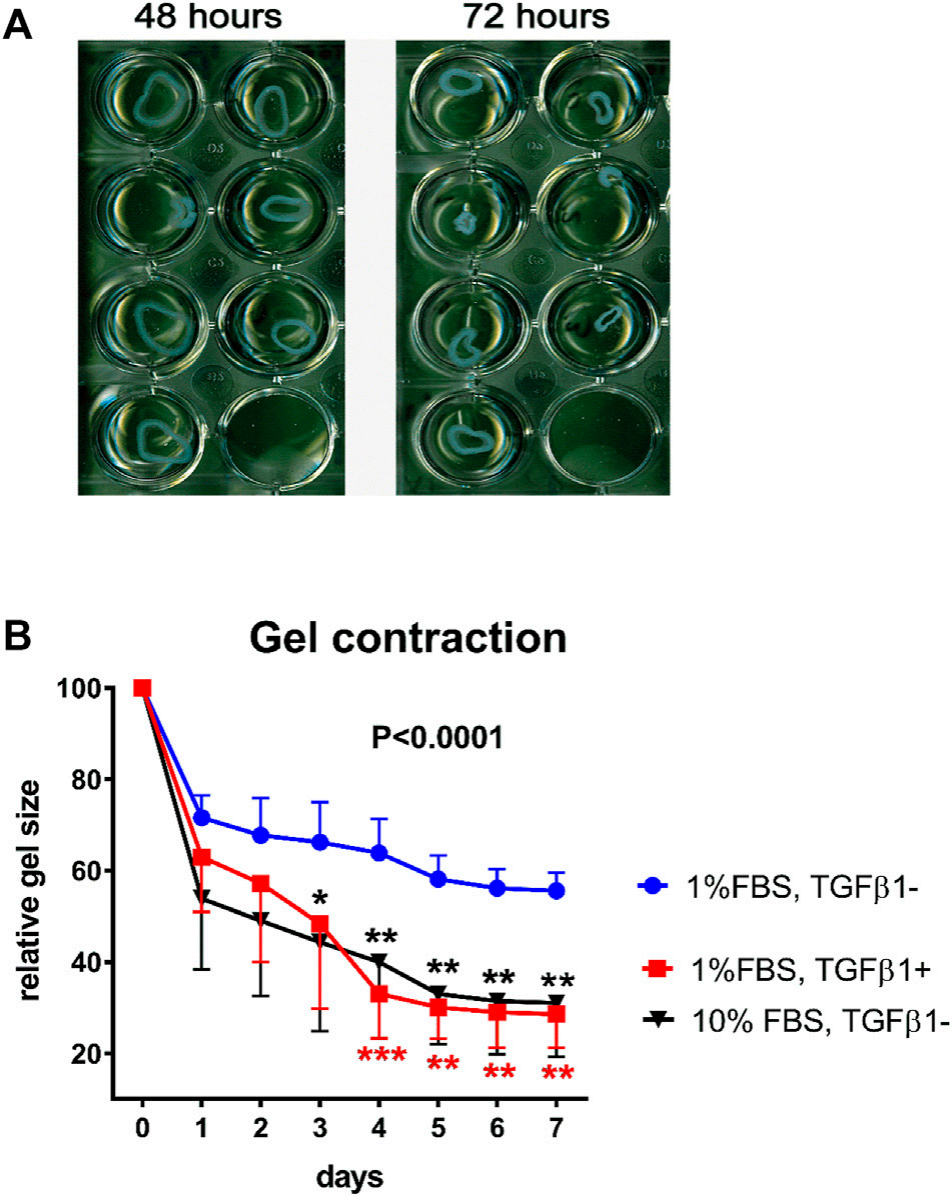
Valve interstitial cells in 3D collagen cultures. Contraction of collagen gel containing cultured interstitial cells from calcified aortic valves (n = 4) and treated with 1% FBS with or without TGF-β1 over a period of 7 days. Panel **(A)** shows how the cultures contract and become smaller. Panel **(B)** shows the collective data of gel contraction of VIC from healthy aortic valves (n = 4) under stimulation with low-serum (1% FBS) with or without TGF-β1 or with high-serum (10% FBS) without TGF-β1. Gel sizes on day 0 were considered as 100%. Data were analyzed by two-way ANOVA with repeated measures. Differences between treatments with 1% FBS with TGF-β1 (shown in red stars) or 10% without TGF-β1 (shown in black stars) compared to 1% FBS without TGFβ1 were determined with Sidak’s multiple comparison post-test, *indicates 0.01 < *p* ≤ 0.05, ** indicates 0.001 < *p* ≤ 0.01, *** indicates 0.0001 < *p* ≤ 0.001. Values are expressed as mean ± SD. Overall *p*-value from two-way ANOVA is shown in bold.

**FIGURE 7:**
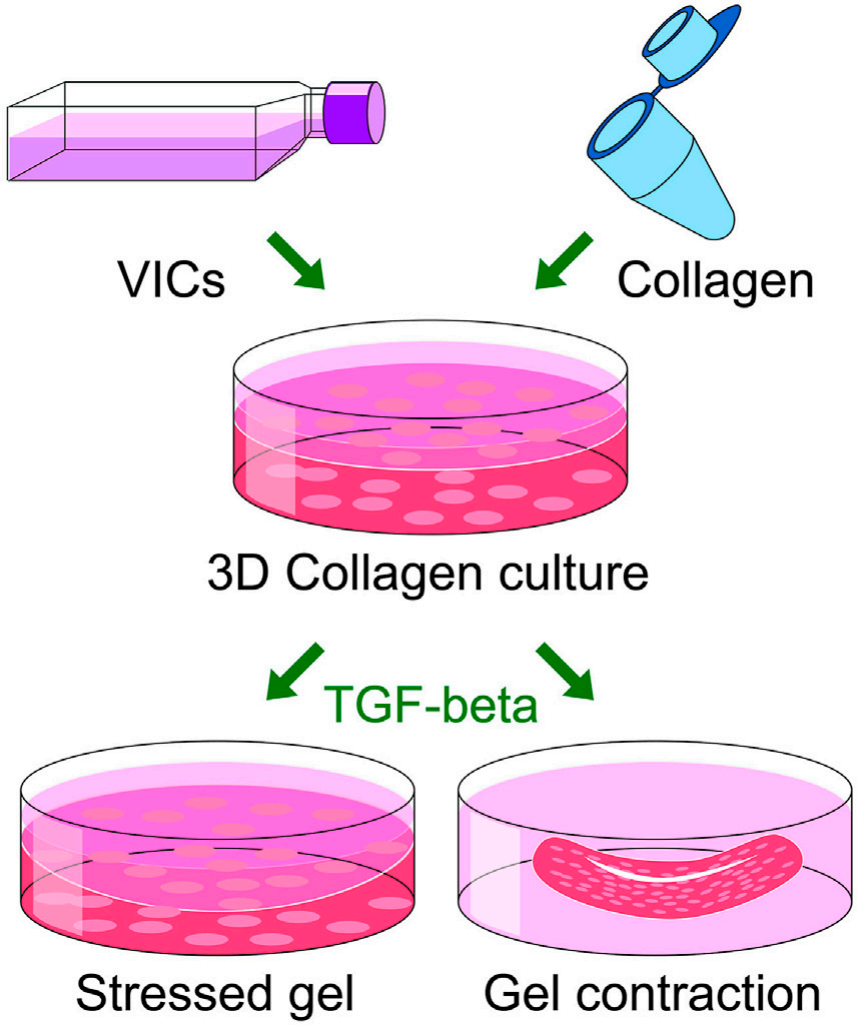
A schematic overview of two 3D cell culture system (stressed gel and gel contraction), based on collagen gel that are being used for measuring the contractility of VIC-derived myofibroblasts.

The collagen gel constructs, in which the VIC are encapsulated (Cushing et al., 2008), may be created with 2 mg/ml collagen I, 5x DMEM (10% FBS 0.1M NaOH) before VIC are added. After polymerization the gels can be gently detached from the wells (floating model), otherwise the gels are kept attached to the well (stressed model). Whereas the floating model is believed to mimic normal connective tissue, stressed model mimics wound healing situation where cells are under mechanical load transferred from extracellular matrix. To stimulate the human VIC to differentiate into myofibroblasts, the gels containing the cells are treated with DMEM supplemented with 1% FBS and 5 ng/ml TGF-β1. Imaging of floating collagen gels are acquired every 24 h (Figure 6A). Collagen gel size is measured and percent contraction is calculated as the change in area from the initial area at time zero. Using this model, we have shown that collagen cell constructs from healthy valves contracted more strongly than if cells were from calcified valves after stimulation with TGF-β1, suggesting higher potential to differentiate into myofibroblasts (Fletcher et al., 2021). A schematic overview of the gel contraction and stressed model formation is shown in Figure 7. Although treatment with 10% FBS does not change expression of myofibroblastic marker αSMA in human VIC compared to treatment with 1% FBS, we noticed that treatments with 10% FBS or 1% FBS without TGFβ1 have different effects on gel contractility of human VIC (Bogdanova et al., 2018). A possible explanation is that serum contains factor(s) that can promote myofibroblast contraction (Parizi et al., 2000; Latif et al., 2015a; Porras et al., 2017). Treatment of floating collagen gel constructs with high-serum (10% FBS) leads to significantly greater collagen gel contractility compared to low-serum (1% FBS) and contracted collagen gel at the same level as stimulation by low-serum (1% FBS) together with TGFβ1 (Figure 6B). In conclusion, collagen gel contraction is a relevant method to characterize functional attributes of human VIC. However, when results are interpreted, it is important to take into consideration factors discussed above that influences collagen gel contractility.

### 5.5 Genetic Modification of Valve Endothelial and Interstitial Cells

**FIGURE 8:**
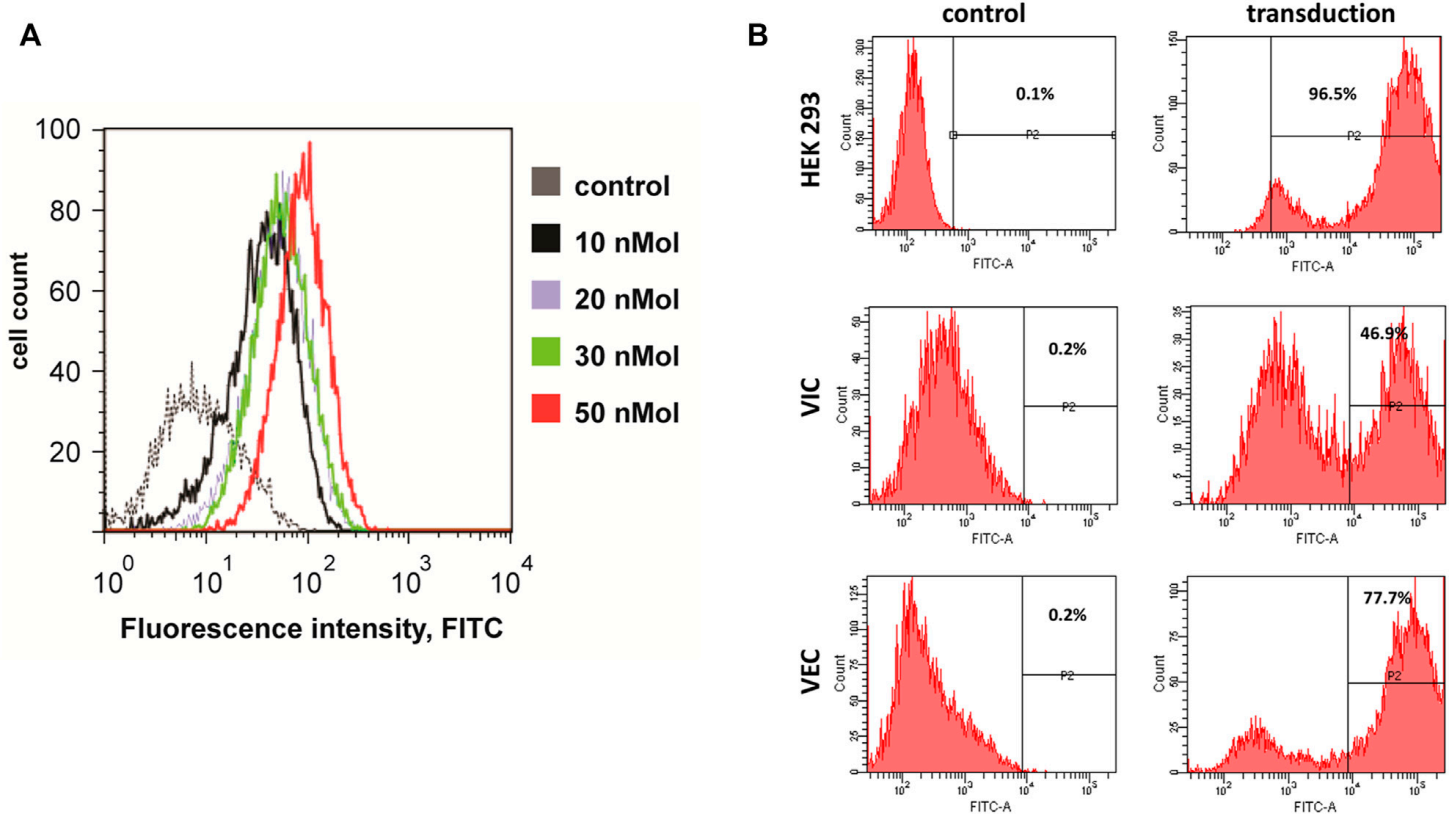
Genetic engineering of valve endothelial (VEC) and interstitial cells (VIC). **(A)** Representative example of fluorescence - activated cell sorting (FACS) analysis of VIC from a calcified aortic valve and transfected with siRNA to mRNA conjugated with fluorescein isothiocyanate (FITC). Transfection kit was used with different concentrations of nanoparticle formulation solution (NFS). Dotted line - negative control (transfected with scrambled siRNA), black line–transfected with 10 nM NFS, blue–20 nM NFS, green -30 nM NFS, red -50 nM NFS (86.5% of cells transfected with siRNA). **(B)** Representative example of fluorescence–activated cell sorting (FACS) analysis of human embryonic kidney cells 293 (HEK293), aortic valve interstitial and endothelial cells from a calcified aortic valve without transduction or transduced with lentiviral construct that encoded green fluorescent protein (GFP).

Both VEC and VIC can be genetically engineered providing tools for unraveling the underlying mechanisms of calcification. We have tested two main gene delivery approaches: 1. Transfection of siRNA using N-TER Nanoparticle delivery system, and 2. Transduction with lentivirus. For the siRNA transfection we assessed the ability of VIC to take up a FITC conjugated siRNA, providing a convenient way to monitor efficiency. Both 10% FBS and serum-free approaches can be used for N-TER Nanoparticle delivery into VIC. Serum free conditions in our hands provided the highest efficiency of transfection with minimal cell death (Figure 8A). VEC appeared to be more susceptible than VIC to lentiviral entry, with approximately 77% of cell being transduced as assessed by GFP expression, while VIC had a transduction efficiency of 47% in comparison to HEK293 (Figure 8B).

## 6 Animal Models

There is generally a lack of animal models that accurately reflect human aortic valve stenosis (Sider et al., 2011). However, animal models are needed to investigate any kind of cardiovascular and soft tissue calcification. In particular to evaluate the effects as well as toxicity of drugs that potentially can inhibit calcification.

### 6.1 Subcutaneous Implantation of Cusps

One extensively used model to study calcification, is implantation of cusp tissue–or other biological materials - in a subcutaneous pouch of rats or rabbits (Fishbein et al., 1982; Levy et al., 1983; Schoen et al., 1985; Mako and Vesely, 1997). This model has been used to evaluate how different preservation techniques influence calcification in cusps of bio-prosthetic heart valves, but also in other biological materials used for implantation, such as pericardial patches. Calcification develops in about 8 weeks when the material tested is explanted (Kennedy et al., 2009; Richards et al., 2013; Gould et al., 2014; Hulin et al., 2018). This model is something between an *in vitro* and *in vivo* model and it is easy to prepare. Although being an un-physiological model, it is suitable for studying inhibition of calcification.

### 6.2 Aortic Valve Leaflets in Culture

As a more complex model than cell culture, culturing aortic valve leaflets may be a good alternative. In the model hierarchy it brings the investigation one step up from the cell cultures. Unfortunately, this is a model where the use of human tissues is less feasible. Healthy human valves are difficult to obtain, and culturing calcified valves may cause problems of interpretations for analysis of calcification. One possibility is to use parts of explanted calcified valves without macroscopic calcification; another possibility would be to use autopsy material. However, the most common practice is to use porcine aortic valve leaflets, either as parts of leaflets or as whole leaflets in culture medium (Sauren et al., 1983; Xing et al., 2004; Konduri et al., 2005; Balachandran et al., 2006; Chester et al., 2008; El-Hamamsy et al., 2009). Most of these studies have focused on the mechanical, biological or contractile properties of valve leaflet tissue. The best results are achieved with pig leaflets which better reflect the human morphology than mice and rat leaflets (Hinton et al., 2008). Rodent valves are also smaller and provide less material for molecular analysis. Several studies induced calcification in pig aortic leaflets. In one, calcification was induced by cyclic stretch for 2 weeks combined with a high concentration of osteogenic medium (Balachandran et al., 2010). Including mechanical stress may add some similarities to the human situation. In another, *Rathan et al.* induced calcification in porcine aortic leaflets by adding phosphate plus inorganic pyrophosphatase for 8 days (Rathan et al., 2014). Chester et al. (2021) developed another model with whole leaflets where calcification is not induced by osteogenic media, but uses the combination of lipopolysaccharide and inorganic phosphate, to initiate and drive the calcification process by an inflammatory response. One of the advantages is the extensive histological investigation of the calcifying leaflets–both qualitative and quantitative (Chester et al., 2021).

#### 6.2.1 A Novel Model of Calcification *ex vivo* in Whole Valve Leaflets

**FIGURE 9:**
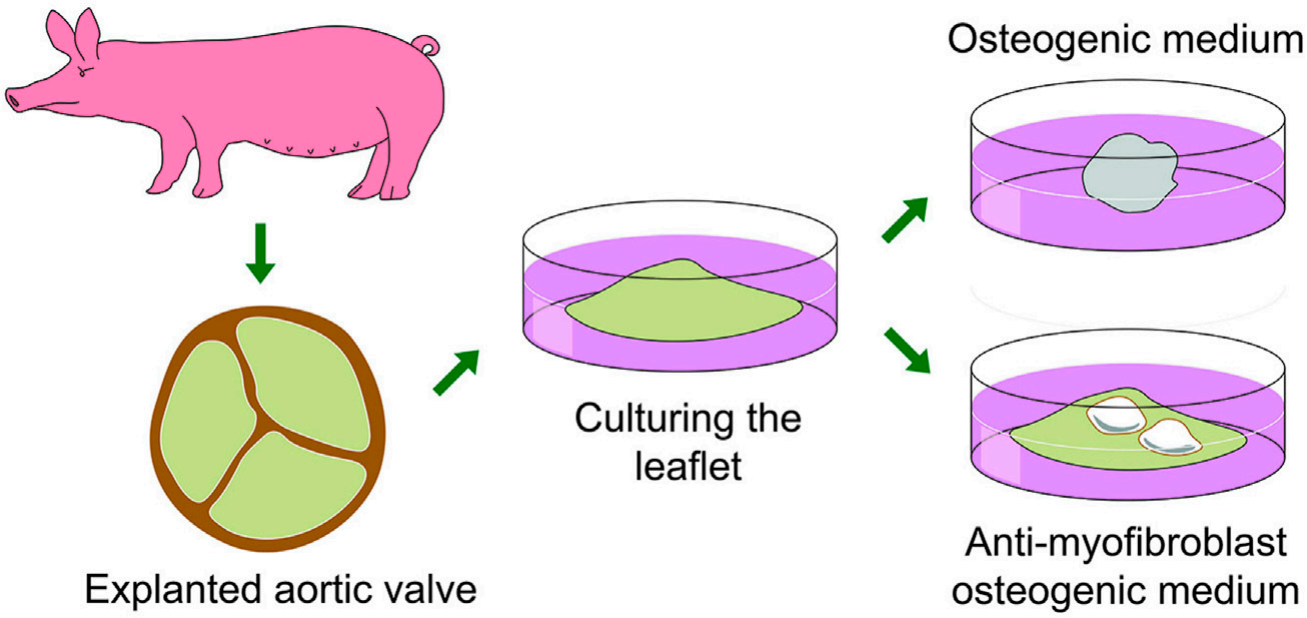
A schematic overview of the whole pig leaflet cultivation and calcification model with the effect of standard osteogenic and anti−myofibroblast media being used.

We have developed a reliable model of cultured whole leaflets from porcine valves (Zabirnyk et al., 2020). Shortly, after animal sacrifice in an authorized abattoir, the hearts are transported on ice to the laboratory where the aortic valve leaflets are dissected free. The whole leaflets are maintained in individual wells of low attachment cell culture plates to avoid cell migration and loss of leaflet integrity. In standard pro-osteogenic media the leaflets shrunk from a leaflet to a ball-like shape due to myofibroblast contraction and with negligible accumulation of calcium deposits. Using anti-myofibroblastic growth medium (low-glucose DMEM, 2% FBS, FGF2 (fibroblast growth factor 2) and insulin), pro-osteogenic stimulation caused strong accumulation of calcium. This formulation prevented leaflets from shrinking, probably by inhibiting myofibroblastic transition of VICs. A schematic overview of the above described culturing pig leaflets in two different growth medias is presented in Figure 9.

The validity of the *ex vivo* leaflet models is solely based on the amount of calcium accumulated in the valves. A limitation is that these leaflet models cannot be expected to closely mimic calcification in patients, a process slowly developing over several years. Histological characterization of the leaflets would be valuable. The *in vitro* leaflets cannot obtain the structure of calcified human valves that have ingrowth of vasculature and containing inflammatory cells and bioactive substances derived from the blood stream plus fibrosis. However, what determines the stiffness of a leaflet giving rise to aortic stenosis is fibrosis and the amount of calcification/calcium in the valve.

#### 6.2.2 Measurements of Calcium in Leaflets

**FIGURE 10:**
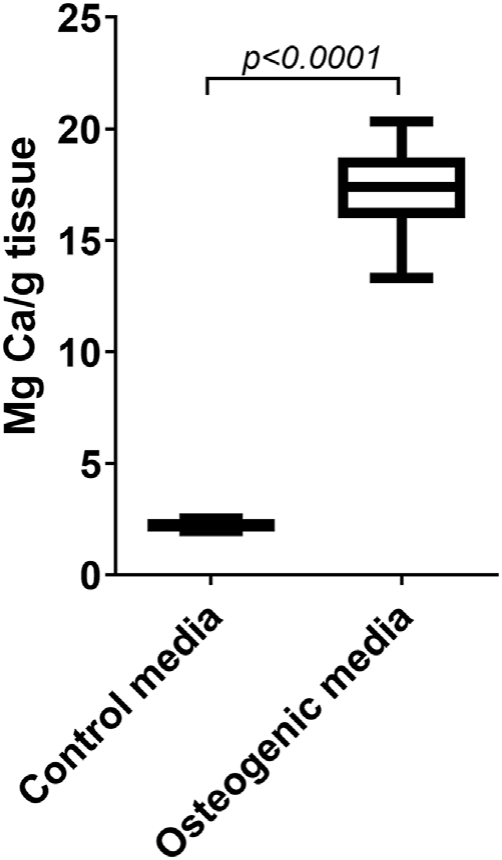
Induced calcification in porcine whole leaflets cultivated *ex vivo*. Calcium accumulation in whole pig leaflets cultivated for 4 weeks in control growth media and in osteogenic media. The amount of calcium was measured by inductively coupled plasma optical emission spectroscopy. The data are shown as box plots and wiskers with 5–95% percentiles.

After the cultivation for 4 weeks with osteogenic differentiation, the amount of calcium accumulation is assessed. Alizarin Red staining is a good method in cell cultures, but it is not suitable as the multilayer tissue nonspecifically absorbs the dye. One way is to section the leaflet and semi-quantitatively assess the regions stained with Alizarin Red, however this method is rather inaccurate. After comparing several methods of quantifying calcium accumulation, the ICP-OES or ICP-MS appeared to be the most reliable and accurate method in whole leaflet models after tissue digestion. An example of calcium accumulation in the leaflets cultivated in growth and osteogenic media measured by ICP-OES is shown in Figure 10.

### 6.3 Animal Models *in vivo*

As already stated above animal models are a necessary instrument for studying the underlying mechanisms of disease and its treatment. Unfortunately, CAVD is a disease with an unmet need for good animal models despite numerous proposed. The most commonly used animals for modelling CAVD are mice, rat, rabbit and porcine, however, only the latter is able to develop CAVD spontaneously (Sider et al., 2011). Below we have provided an overview of the most commonly used animal models.

#### 6.3.1 Mouse Models

The majority of animal models of CAVD have been developed in mice. This is because of their cost-efficiency, rapid breeding and, most importantly, the availability of genetically modified variants. Regrettably, mouse models have significant limitations. Neither mouse nor rat aortic valve leaflets have the tri-layer structure akin to the human leaflet, only several layers of cells (Hinton et al., 2008). Wild-type mice do not develop aortic valve stenosis, however, a diet-based model has been reported with mild to moderate aortic stenosis (Drolet et al., 2006). A better alternative is transgenic mouse models. Until recently, the most commonly used mouse models contained a single gene mutation which affected lipid metabolism, the low-density lipoprotein receptor (*Ldlr* ^*−/−*^) and apolipoprotein E deficient mice (*ApoE* ^*−/−*^). These mice developed significant aortic valve calcification and some signs of CAVD when fed a high cholesterol diet. However, these models do not develop hemo-dynamically significant aortic valve stenosis (Rajamannan, 2014). To achieve stenosis the complex hypercholesterolemic mouse model with mutations in both Ldlr and ApoB100 (Apolipoprotein B100) (*Ldlr* ^*−/−*^ */ApoB* ^*100/100*^ mice) is necessary. It is efficiency is significantly increased if fed with a high cholesterol Western diet over 12 months (Weiss et al., 2006; Miller et al., 2009; Miller et al., 2010). This model is further developed by addition of a conditional knockout of the microsomal triglyceride transfer protein (*Mttp*) which plays a critical role in production of apolipoprotein B-containing lipoproteins (*Ldlr−/−*/*Apob* ^100/100^/*Mttp* ^fl/fl^/*Mx1-Cre* ^+/+^)–the so-called Reversa model. This allows controlled onset of hyperlipidemia during the experimental aortic valve stenosis development (Miller et al., 2009). A recent study reports an improved of *Ldlr* ^*−/−*^ */ApoB* ^*100/100*^ mouse model that develops aortic stenosis earlier − after 6 month with high fat diet treatment - and gives insight into the role of platelet-derived TGF-β1 in CAVD progression (Varshney et al., 2019).

Several non-hyperlipidemic models offer features of CAVD including aortic valve leaflet calcification, but they lack the development of aortic stenosis. They include mice containing mutations in MGP (Matrix Gla protein) (Luo et al., 1997), EGFR (Epidermal growth factor receptor) (Barrick et al., 2009), Klotho (Cheek et al., 2012), RBPJk (Recombination Signal Binding Protein For Immunoglobulin Kappa J Region) (Nus et al., 2011) and IL1RN (Interleukin 1 Receptor Antagonist) (Isoda et al., 2010). Interestingly, despite strong calcification, the combination of high fat diet and vitamin D supplementation does not enhance the aortic stenosis phenotype of the EGFR mouse (Colleville et al., 2019).

Some genetic mouse models resemble human congenital aortic valve defects, which present with increased occurrence of CAVD. Bicuspid aortic valves are reported in mice containing mutations in eNOS (Endothelial nitric oxide synthase) (Lee et al., 2000), Notch1(Notch Receptor 1) (Nigam and Srivastava, 2009), Postn (Periostin) (Tkatchenko et al., 2009). A unicuspid aortic valve with some signs of CAVD was reported in a novel mouse model heterozygous for a dominant loss-of-function mutation in EGFR (*Egfr* ^Vel/+^) (Weiss et al., 2018).

In addition to dietary and genetic mouse models, an *in vivo* valve injury model was developed by insertion of a spring guide wire into the left ventricle via the right common carotid artery under echocardiographic guidance, and scratching the leaflets with the body of the wire (Honda et al., 2014). This model was recently improved to achieve either mild, moderate or severe cusp injury to enable a more reproducible study of different stages of CAVD (Niepmann et al., 2019). It is important to notice that this direct injury models demonstrate typical clinical features of CAVD including inflammation, valve thickening, fibrosis and calcification combined with hemo-dynamically significant aortic stenosis as well as regurgitation (in severe injury). This model together with the *Ldlr*-deficient, *ApoB100*-only mice (*Ldlr* ^*−/−*^ */ApoB* ^*100/100*^) model appears to be the most relevant murine models of CAVD to date.

#### 6.3.2 Rat Models

The rat aortic valve leaflets like the mouse, are not optimal for studies on CAVD because they consist of several cell layers without the tri-layered structure of human aortic valves (Grauss et al., 2003) (see above). A common model of vascular calcification and CAVD in rats is based on intravenous treatment with Warfarin, however, these rats do not develop hemo-dynamically significant aortic stenosis (Price et al., 1998) and warfarin-induced aortic valve calcification differs from the naturally occurring (Venardos et al., 2022). Such rats are phenotypically similar to MGP mutant mice, suggesting similar underlying mechanisms (Tsang et al., 2016). Mirroring the fact that renal failure is also associated with CAVD in humans, several uremic rat models induced by nephrectomy or high-adenine diet develop aortic valve calcification (Shuvy et al., 2008; Roosens et al., 2013a; Roosens et al., 2013b). Furthermore, vitamin D treatment causes vascular and aortic valve calcification in rats, but without aortic stenosis (Roosens et al., 2011). All taken together, rats do not represent an appropriate experimental model of aortic valve calcification with aortic stenosis.

#### 6.3.3 Rabbit Models

Rabbits have both advantages and disadvantages as model for CAVD. They have the tri-layer leaflet composition similar to humans, several similarities in lipoprotein metabolism, and natural mutant and transgenic strains are available. Most frequently a hypercholesterolemic diet is administered to cause CAVD (Guerraty and Mohler Iii, 2007). A 40-weeks treatment with such diet induces early development of aortic stenosis (Cimini et al., 2005). When a hypercholesterolemic diet was coupled with vitamin D-induced hypercalcemia, significant calcium deposition developed in addition to aortic stenosis (Drolet et al., 2003; Guerraty and Mohler Iii, 2007). Another study demonstrated that a hypercholesterolemic and vitamin D2-supplemented diet caused leaflet thickening, calcification, matrix disorganization, and aortic stenosis (Marechaux et al., 2009). This combination appears to provide a better model of CAVD than hypercholesterolemic or vitamin D diets alone (Roosens et al., 2013a). However, a rabbit model using high-cholesterol diet is limited by liver dysfunction and high mortality rates due to cholesterol overload (Hara et al., 2018). In contrast, rabbit genetic models that have alterations in the *Ldlr* and/or apolipoprotein-encoding genes result in hypercholesterolemia even under a cholesterol-free, limited fat diet without cholesterol overload (Sider et al., 2011). Such a model is the Watanabe heritable hyperlipidemic rabbits that develop valve thickening, calcification, aortic stenosis and calcification-related gene activation (Hara et al., 2018). In addition to the hypercholesterolemic models, a hypertensive rabbit model develops increased valve thickness and mild aortic stenosis (Cuniberti et al., 2006).

#### 6.3.4 Pig Models

The pig has tri-layered aortic valve leaflets similar to humans. Unlike mouse, rat and rabbit models, pigs are prone to naturally develop valvular atherosclerotic lesions (Skold et al., 1966). Swine develop valvular lesions and early signs of CAVD when fed with a high-fat/high-cholesterol diet for 5 months (Sider et al., 2014). Aortic valve calcification has been shown to be restricted to the aortic side in early CAVD in a porcine models with hypercholesterolemic diet (Guerraty et al., 2010). The Rapacz-familial hypercholesterolemic swine mutants develop leaflet thickening, increased lipid oxidation and infiltration of macrophages, however, further stimulation is needed to develop more advanced stages of CAVD with aortic stenosis (Porras et al., 2015). Additional swine models with lipid metabolism mutations used in atherosclerosis research may have a potential to be used in CAVD research (Sider et al., 2011). Tsang *et al.* have published a detailed review on the pig as a model for cardiovascular disease including CAVD (Tsang et al., 2016).

#### 6.3.5 Other Animal Models

There are other animal models available, although potentially useful, they are not commonly used. For example, naturally occurring and experimental aortic stenosis has been investigated in dogs (Copeland et al., 1974; Kim et al., 1986; Ahlstrom Ast et al., 2008). Sheep are routinely used as a big animal model to investigate calcification of biological aortic valve prosthesis and homografts *in vivo* (Kheradvar et al., 2017; Theodoridis et al., 2017; Bester et al., 2018). Apparently, calcification occurs very rapidly in sheep compared to humans.

## 7 Multiomnics

### 7.1 Proteomics as an Example of Multi-Omnics Approaches

Multi-omnic approaches with proteomics, metabolomics and transcriptomics have recently gained momentum in aortic valve calcification investigations (Schlotter et al., 2018). Here as an example we have listed several approaches to perform proteomics analysis in CAVD research. Several groups have targeted proteome changes in human plasma during the development of calcific aortic valve disease for better understanding the basic mechanisms and to discover biomarkers (Gil-Dones et al., 2012; Satoh et al., 2015; Mourino-Alvarez et al., 2016; Olkowicz et al., 2017; Ljungberg et al., 2018). Additionally, gaining access to the plasma of both CAVD patients and healthy controls is rather straight-forward. This approach may be useful for the identification of biomarkers of CAVD in the blood of patients. The later aim is especially important because of the current lack of screening for early detection of CAVD. Targeting known problems of the proteome complexity in plasma, Gil−Dones *et al.* (Monzack and Masters, 2011) suggested improved protocols for plasma proteomics analysis in CAVD research.

An *ex vivo* modification of the plasma proteome analysis in patients with calcified aortic valves was reported as a secretome proteomics analysis from the explanted whole human leaflets kept for some time in growth media (Alvarez-Llamas et al., 2013; de la Cuesta et al., 2013). This approach allows one to mimic the secretome entering the circulation from aortic leaflets without interference from other tissues. Another common approach is to perform proteomics on the whole human leaflets explanted during the surgery or autopsy after lysate of the valve leaflets (Martin-Rojas et al., 2015; Weisell et al., 2019). A protein extraction procedure optimization was reported for this approach (Gil-Dones et al., 2010). The use of more advanced proteomics technique such as MALDI-imaging mass spectrometry offers the advantage to investigate the pathophysiological changes taking place in calcified aortic valves while retaining the histopathological context. This allows the simultaneous mapping of hundreds of peptides and proteins present in tissue sections with a lateral resolution of approximately 50–75 microns (Martin-Rojas et al., 2015).

Direct analysis of whole leaflets explanted from humans is most relevant to *in vivo* assessment, however, it has an important drawback as the analysis is performed on all cell types within the valve. Several groups partly overcome this problem by performing macro- (Matsumoto et al., 2012; Suzuki et al., 2016) and microdissection (Schlotter et al., 2018), subdividing the valve into calcified and non−calcified regions.

Another approach is to isolate and propagate *in vitro* VICs and subsequently perform proteomic analysis on human (Yu et al., 2018; Goto et al., 2019), bovine (Renato et al., 2013) or rat (Cui et al., 2017) cell cultures. Some authors have reported clonogenic sub−fractioning of the isolated and cultivated bovine VIC prior to proteomics analysis (Bertacco et al., 2010; Rattazzi et al., 2020). Unfortunately, gene studies in cultured VIC are influenced by the culture process *per se*. At the same time, omics analysis of calcified whole leaflets are “impure” containing material from several cell types in addition to VIC: VEC, vascular cells including smooth muscle cells from vascular ingrowth, as well as macrophages and other inflammatory cells. A combination of the above-mentioned proteomics approaches (whole leaflet, secretome, cell cultures, and plasma proteomics analysis) reveals more data than individual approaches (Martin-Rojas et al., 2017). Microarray and RNA sequencing with transcriptomics, in particular if combined with proteomics, might provide valuable information about signaling of the calcification process.

## 8 Biomarkers of Aortic Valve Calcification

To identify high-risk asymptomatic patients with aortic stenosis has become a major topic of interest during the last years. However, detailed discussion of risks and indications for surgical intervention is beyond the scope of this article (see review by Lindman et al. (Lindman et al., 2020)). Among a jungle of advanced and sometimes costly imaging modalities which may be predictive of disease progression and mortality in aortic stenosis (Nchimi et al., 2018), a blood sample for measuring circulating biomarkers is a simple, inexpensive, and easily available method to provide information about the stage and possible risks of asymptomatic aortic stenosis. Even if biomarkers represent indirect assessment, they might possibly be helpful to identify progression of CAVD and asymptomatic patients who then would benefit from aortic valve replacement.

Most interest has been focused on natriuretic peptides, in particular brain-natriuretic peptide (BNP) and its pro-hormone N-terminal pro B-type natriuretic peptide (NT-proBNP) as possible biomarkers of aortic stenosis (Weber et al., 2004; Steadman et al., 2010; Clavel et al., 2014; Auensen et al., 2017; Small et al., 2017). The biomarker does not reflect calcification *per se*, but it provides diagnostic and prognostic information about myocardial remodeling as a consequence of aortic stenosis. Marked increased levels of BNP may reflect irreversible injury to the myocardium and has been shown to predict worse outcome in patients after transcatheter aortic valve interventions (O'Neill et al., 2015). The same is the case with cardiac troponins (Köhler et al., 2016). BNP is the only biomarker in the circulation accepted to have prognostic value in the guidelines of the European Society for Cardiology and the European Association for Cardio-Thoracic Surgery (but not for the American Heart Association or the American College of Cardiology).

Recently, a series of other potential biomarkers in the circulation have brokered interest, such as for instance von Willebrand Factor (vWF) due to high shear stress in aortic stenosis (Van Belle et al., 2019). Plasma levels and function of vWF is reduced in parallel with severity of aortic stenosis. The levels are normalized after transcatheter aortic valve intervention, but did not normalize if a paravalvular leakage was present (Van Belle et al., 2016).

Of particular interests for this review are biomarkers that may be directly related to the calcification process. This includes microRNAs (Oury et al., 2016), fetuin-A (Di Minno et al., 2017), osteopontin (Sainger et al., 2013), osteoprogeterin (Ueland et al., 2011), and MGP (Ueland et al., 2010). Notch may have an important role in aortic valve calcification (Kostina et al., 2018) and the Notch ligand Delta-1 is elevated and associated with mortality in patients with symptomatic aortic stenosis (Abraityte et al., 2015). Elmariah *et al.* suggested that a panel of multiple biomarkers including age, NT-proBNP, vWF, and fetuin-A would be valuable for the identification of high-risk patients with aortic stenosis and for timely valve intervention (Elmariah et al., 2018). MacGrogan *et al* also suggested that a set of several genes in blood provided a “gene signature” predicting aortic valve calcification (MacGrogan et al., 2020).

So far neither guidelines of the American Heart Association, the American College of Cardiology, the European Society of Cardiology, nor the European Association for Cardio-Thoracic Surgery include these biomarkers as valuable for evaluation of patients with CAVD. The role of biomarkers as a guide to more aggressive aortic valve replacement in asymptomatic patients has yet to be investigated. It might well be in the future a profile of several biomarkers may be useful. A full discussion of the field is beyond the scope of this review, however, several concise and recent reviews have been published on this topic (Redfors et al., 2017; Small et al., 2017; Patel and Kumbhani, 2018; Toutouzas et al., 2019; Oury et al., 2020).

## 9 Calcium Phosphate Protein Particles

Circulating calcium phosphate protein particles might be important both for the understanding of the processes leading to calcification and for the development of therapy for both valvular and vascular calcifications. Such particles have not been found in the circulation of healthy individuals, but exist in the circulation of patients with some inflammatory diseases (Smith et al., 2013). The number of particles in the blood can be reduced by sodium thiosulphate which has been suggested to reduce vascular calcification (Cai et al., 2013). Fetuin-A is a key player in the formation of calcium phosphate protein particles. This protein is an endogenous inhibitor of soft tissue calcification by inhibiting formation of calcium phosphate (Heiss et al., 2010). Once a mineral nuclei is formed, fetuin-A binds to the apatite surface and inhibits the formation of larger entities (Price and Lim, 2003). The nanoparticles consisting of calcium phosphate crystals may be a way to clear calcium and inhibit calcification; they are cleared from the circulation in the liver and the spleen, a process which is dependent on scavenger receptors on phagocyte surfaces (Herrmann et al., 2012). The role of calcium phosphate protein particles in soft tissue calcification is uncertain, however, in pro-calcific situations, the particles may have structural transformation into larger particles with a crystalline core and initiate calcification (Jahnen-Dechent et al., 2011). Using nano-analytical electron microscopy techniques, Bertazzo *et al.* found such mineralized particles on the aortic valve even before calcification of the valve (Bertazzo et al., 2013). The presence of these particles might perhaps even initiate CAVD (Bertazzo and Gentleman, 2017). This is in line with findings that crystallinity of hydroxyapatite in 3D cultures with VEC and VIC increase calcium accumulation (Richards et al., 2018). Detailed methods for studying calcium phosphate particles in human serum, on tissues, and in tissues include ultracentrifugation, gel filtration, scanning and transmission electron microscopy, measurements of calcium and phosphate, energy-dispersive X-ray spectroscopy, selected area electron diffraction analyses, and material science technology in general (Price and Lim, 2003; Bertazzo et al., 2013).

## 10 Discussion

The process of aortic valve calcification is still far from elucidated. In this overview we try to cover presently used methods to study CAVD, from translational studies in cell cultures to patient studies. With the lack of good animal models, translational studies in cell cultures are by far the most frequently used model to clarify the cellular and molecular mechanisms of calcification. Consequently, this is the only model where more detailed techniques were presented. Cells from human aortic valves should be used in order to avoid species differences. Cell models are also suitable for screening of potentially inhibitory drugs. There is an unmet need for good models of aortic valve calcification in animals where the structure of the valve leaflet is similar to the structure of human aortic valve. Moreover, we know too little about the mineral structure of calcified valves including its role. With increasing use of endovascular implantation of aortic valve prostheses, good imaging of the aortic ostium and the valve has become more and more important. Possibly, MRI should be used more extensively. There is also a need for good biomarkers. Unfortunately, although there are suggestions for several biomarkers, it is highly uncertain how they should be used. Biomarkers cannot replace imaging because the structure and degree of stenosis are decisive for clinical decisions.
